# Calcium oxalate crystals induces tight junction disruption in distal renal tubular epithelial cells by activating ROS/Akt/p38 MAPK signaling pathway

**DOI:** 10.1080/0886022X.2017.1305968

**Published:** 2017-03-24

**Authors:** Lei Yu, Xiuguo Gan, Xukun Liu, Ruihua An

**Affiliations:** a Department of Urology, the First Affiliated Hospital of Harbin Medical University, Harbin, Heilongjiang Province, P.R. China;; b Department of General Surgery, the People's Hospital of Jixi, Jixi, Heilongjiang Province, P.R. China

**Keywords:** Calcium oxalate crystals, tight junction, ZO-1, ROS, Akt, p38 MAPK

## Abstract

Tight junction plays important roles in regulating paracellular transports and maintaining cell polarity. Calcium oxalate monohydrate (COM) crystals, the major crystalline composition of kidney stones, have been demonstrated to be able to cause tight junction disruption to accelerate renal cell injury. However, the cellular signaling involved in COM crystal-induced tight junction disruption remains largely to be investigated. In the present study, we proved that COM crystals induced tight junction disruption by activating ROS/Akt/p38 MAPK pathway. Treating Madin–Darby canine kidney (MDCK) cells with COM crystals induced a substantial increasing of ROS generation and activation of Akt that triggered subsequential activation of ASK1 and p38 mitogen-activated protein kinase (MAPK). Western blot revealed a significantly decreased expression of ZO-1 and occludin, two important structural proteins of tight junction. Besides, redistribution and dissociation of ZO-1 were observed by COM crystals treatment. Inhibition of ROS by *N*-acetyl-l-cysteine (NAC) attenuated the activation of Akt, ASK1, p38 MAPK, and down-regulation of ZO-1 and occludin. The redistribution and dissociation of ZO-1 were also alleviated by NAC treatment. These results indicated that ROS were involved in the regulation of tight junction disruption induced by COM crystals. In addition, the down-regulation of ZO-1 and occludin, the phosphorylation of ASK1 and p38 MAPK were also attenuated by MK-2206, an inhibitor of Akt kinase, implying Akt was involved in the disruption of tight junction upstream of p38 MAPK. Thus, these results suggested that ROS-Akt-p38 MAPK signaling pathway was activated in COM crystal-induced disruption of tight junction in MDCK cells.

## Introduction

Kidney stone disease is caused by precipitation and retention of poorly soluble salts in the kidney, whose recurrence rate is approximately 40% at 5 years after the first treatment.^1^ Calcium oxalate monohydrate (COM) is the major crystalline composition of kidney stone matrix, accounting for up to 70%.^2^ Adhesion of COM crystals to renal tubular epithelial cell is a crucial mechanism for kidney stone formation.^3^^,^^4^ The interaction between COM crystals and renal cells leads to several cellular responses, including overproduction of reactive oxygen species (ROS),^5^^,^^6^ cellular injury,^7^ and final tissue inflammation.^8^ Recently, it has been demonstrated that COM crystals can cause tight junction disruption of renal tubular epithelial cells, accompanied with impairment of its barrier and fence functions^9^ characterized by decreased expression levels, redistribution and dissociation of tight junction structural proteins (ZO-1, occludin, and claudin). However, the cellular signaling pathways activated in renal cells following COM exposure are not well delineated and continue to be a large area of interest to be investigated. It had been reported that p38 mitogen-activated protein kinase (MAPK) activation was involved in COM crystals induced tight junction disruption in distal renal tubular epithelial cells.^10^ However, more detailed molecular mechanisms besides p38 MAPK activation in COM-induced tight junction disruption remain to be elucidated.

COM crystals–cell interactions lead to the production of ROS, which can trigger epithelial cell injury, inflammation, and ultimately result in cell apoptosis or death.^11^^,^^12^ ROS, such as hydrogen peroxide (H_2_O_2_), are generally small, short-lived, and highly reactive molecules, which play an important role in the regulation of cell signaling pathways involved in proliferation, apoptosis, and senescence.^13^ An aberrant increase in the level of ROS can damage nucleic acids, proteins, and intracellular membranes, which lead to oxidative stress and impairment of cell structures and functions.^14^ The oxidative stress is well known to disrupt the epithelial tight junctions,^15^ and it has been reported that oxidative stress induced by ROS disrupts tight junctions and increases paracellular permeability in a variety of epithelial tissues.^16–18^ Moreover, previous studies have shown that ROS can activate p38 MAPK in the regulation of UVB-induced mitochondrial apoptosis,^19^ nickel compound-induced apoptosis,^20^ palmitic acid-stimulated hepatocyte proliferation,^21^ and γ-ionizing radiation-induced apoptotic cell death.^22^ Thus, as an activator of p38 MAPK, ROS may be involved in the signaling pathway of COM crystal-induced tight junction disruption.

ROS have been reported to be involved in the activation of Akt (Protein Kinase B) signaling pathway.^23^^,^^24^ Akt, serine/threonine kinase, plays critical roles in regulating growth, proliferation, survival, metabolism, and other cellular activities. However, in contrast to its well-established survival-promoting role, it was found that Akt also could induce cell apoptosis^20^^,^^25^ or sensitize cells to senescence or death.^26^ Apoptosis signal-regulating kinase 1 (ASK1) is a serine-threonine kinase, which has been reported to be phosphorylated by Akt at serine 83 (Ser83) or threonine 838 (Thr838), resulting in the reduced or increased activity respectively.^27–29^ ASK1 was initially discovered as a mitogen-activated protein kinase kinase kinase (MAPKKK) in the c-Jun N-terminal kinase/stress-activated protein kinase (JNK/SAPK) and p38 MAPK signaling cascades.^28^^,^^30^ A variety of stimuli can activate ASK1, including TNF-α, ROS, lipopolysaccharide (LPS), and genotoxic stress, and activated ASK1 further activates p38 and JNK via activating the MAP2Ks, MKK4/MKK7 and MKK3/MKK6, leading to cell apoptosis.

Taken together, we hypothesized that COM crystals induced tight junction disruption by activating ROS/Akt/p38 MAPK pathway in distal renal tubular epithelial cells. In the present study, we tested the hypothesis in Madin–Darby canine kidney (MDCK) cells. ROS generation and cell apoptosis were analyzed to determine the effects of COM crystals on tight junction in MDCK cells using flow cytometry. Western blot was performed to explore the expression regulation of the associated proteins lying on the signal pathway of tight junction. Immunofluorescence assay was performed to demonstrate the redistribution and localization alterations of ZO-1. Moreover, *N*-acetyl-l-cysteine (NAC) and MK-2206, the inhibitors of ROS and Akt, were employed to reveal the involvement of ROS and Akt in the regulation of tight junction disruption induced by COM crystals in MDCK cells.

## Materials and methods

### Preparation of COM crystals

COM crystals were prepared by the method described in previous study.^31^ Briefly, 10 mM calcium chloride dihydrate (CaCl_2_·2H_2_O, Sigma-Aldrich, St. Louis, MO) and 10 mM sodium oxalate (Na_2_C_2_O_4_, Sigma-Aldrich, St. Louis, MO) used as stock solution were added to basic buffer 10 mM Tris–HCl (pH 7.4) to make up their final concentration of 5 and 0.5 mM. Then, the mixture was incubated at room temperature overnight followed by centrifugation at 3000 rpm for 5 min. The supernatant was discarded and the precipitation was washed with methanol. Then, methanol was removed after another centrifugation at 3000 rpm for 5 min, and the precipitation was dried overnight at 37 °C. Finally, the precipitation was decontaminated by UV light radiation for 30 min, then used as COM crystals for subsequent experiments.

### Cell culture and treatment

MDCK cells were grown in Eagle’s minimum essential medium (MEM; Thermo Scientific, Grand Island, NY) supplemented with 10% fetal bovine serum (FBS, GE Healthcare, Pittsburgh, PA), 1.2% penicillin-G/streptomycin and 2 mM l-glutamine, and maintained in a humidified incubator at 37 °C with 5% CO_2_. MDCK cells were seeded and incubated in a 6-well or 24-well plate overnight, and treated with COM crystals for 48 h, and then subjected to subsequent investigations. For COM-treated group, COM crystals were added to complete Eagle’s MEM medium to obtain a final concentration of 1 mM. For the pretreatment of two inhibitors, NAC (inhibitor of ROS, Sigma-Aldrich, St. Louis, MO) was added to the complete medium of MDCK cells and incubated for 2 h at the final concentration of 10 mM^20^; and MK-2206 (inhibitor of Akt, Selleck) was added to the medium and incubated for 24 h at the final concentration of 5 μM.^32^ After pretreatment with NAC or MK-2206, MDCK cells were treated with or without 1 mM COM crystals for 48 h.

### Analysis of cell death (Annexin V/propidium iodide double staining)

For cell death assay, MDCK cells were stained with Annexin V-FITC and propidium iodide (PI) according to the manufacturer’s protocol (HaiGene, Harbin, China) and then evaluated for apoptosis using flow cytometry. Briefly, MDCK cells seeded in 60 mm dish (including both adherent and floating cells) were trypsinized with trypsin and resuspended in 2 mL MEM. The harvested cells were centrifuged at 1500 rpm for 5 min, and washed twice with PBS. Cell pellets were resuspended in 1 × Annexin V binding buffer at a final concentration of 2 × 10^5^ cells/mL and then stained with 5 μL of Annexin V-FITC and 8 μL of PI in 1 × Annexin V binding buffer for 10 min at room temperature in the dark. The apoptotic cells were determined using Guava^®^ easyCyte flow cytometry with GuavaSoft 2.5 (Millipore, Darmstadt, Germany). This experiment was performed in triplicate. Percentage of total cell death (% cell death) = (number of both apoptotic and necrotic cells/number of all cells) × 100%.

### Determination of ROS

The production of ROS was monitored using the nonfluorescent probe 2′,7′-dichlorodihydrofluorescein diacetate (DCFH-DA, Sigma-Aldrich, St. Louis, MO). DCFH-DA diffuses into cells and is deacetylated by esterases to form the nonfluorescent product 2′,7′-dichlorodihydrofluorescein (DCFH). In the presence of ROS, DCFH reacts with ROS to form the fluorescent product 2′,7′-dichlorofluorescein (DCF). After treated with COM crystals or NAC, MDCK cells were incubated with DCFH-DA that was pre-diluted to a final concentration of 10 μM for 30 min at 37 °C in the dark. Then, MDCK cells were washed twice with PBS, trypsinized, and resuspended in PBS. Fluorescence was measured at an emission wavelength of 530 nm and an excitation wavelength of 480 nm by flow cytometry (Millipore Guava EasyCyte HT).

### Western blot analysis

The total cellular samples were washed twice with PBS and lysed in RIPA buffer (HaiGene) supplemented with 1 mM PMSF (Sigma-Aldrich, St. Louis, MO). The concentration of total protein was determined using BCA Protein Assay Kit (Pierce, Rockford, IL). The total cellular protein extracts were separated by SDS–PAGE and transferred onto the nitrocellulose membrane (PALL, Washington, NY) in 20 mM Tris–HCl (pH 8.0) containing 150 mM glycine and 20% (v/v) methanol. The membrane was blocked with 5% nonfat dry milk in 1 × TBS containing 0.05% Tween 20 and incubated with primary antibody at 4 °C overnight. Antibodies against Akt (1:1000), phospho-Akt (Ser473) (1:500) were purchased from Cell Signaling Technology. Antibodies against ASK1 (1:500), phospho-ASK1 (Thr845) (1:500), p38 (1:1000), phospho-p38 (Tyr182) (1:500), β-Actin (1:1000) and secondary antibodies (1:5000) were all purchased from Santa Cruz (Weatherford, TX). Occludin antibody (1:1000) was obtained from Invitrogen, and ZO-1 antibody (1:1000) was obtained from Abcam (Cambridge, MA). The signals were developed with enhanced chemiluminescence reagent (HaiGene), and the digital images were captured using a LAS-4000 CCD camera system (Fuji film, Tokyo, Japan). The relative intensity of bands was analyzed by the software of Image-Pro Plus 6.0 (Media Cybernetics, Silver Spring, MD).

### Immunofluorescence staining

MDCK cells (1 × 10^5^) were seeded in a 24-well plate and cultured on the cover slips. After treated with or without NAC or MK-2206 followed by incubating with COM crystals for 48 h, MDCK cells were collected for subsequential immunofluorescence analysis. Briefly, MDCK cells were rinsed with PBS for three times, then fixed with 4% formaldehyde for 20 min, and permeabilized with 0.1% Triton X-100 for 15 min. After blocked with 2% BSA for 1 h, the fixed cells were incubated with primary antibody against ZO-1 (1:50) at 4 °C overnight. After washing for three times with PBS, the cells were incubated with AlexaFluor-488-conjugated secondary antibody (1:100) for 2 h at room temperature. Then, the cell cover slips were mounted with 50% glycerol/PBS after washing with PBS for five times. The cells were visualized and the images were captured using a fluorescent microscopy (Leica, Wetzlar, Germany).

### Statistical analysis

All the experiments were done in triplicate and the results were expressed as mean ± standard deviation (SD). Comparisons of the data among different groups were performed by one-way ANOVA using SPSS software version 13.0 (SPSS Inc., Chicago, IL). *p* < .05 were considered to be statistically significant.

## Results

### Effects of differential doses of COM crystals on cell death

**Figure 1.**
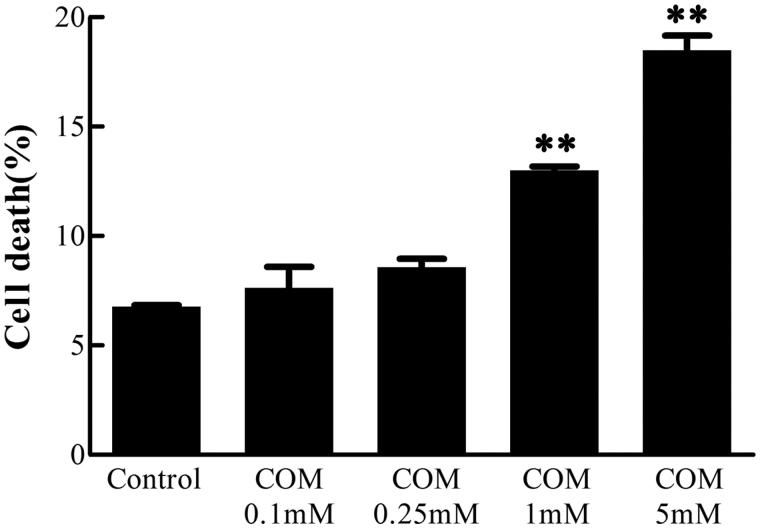
COM crystals-induced cell death assay. MDCK cells were treated with various doses of COM crystals for 48 h, and then subjected to Annexin V FITC/PI co-staining followed by flow cytometry analysis. The percentage of cell death = (the number of apoptotic and necrotic cells/the number of total cells) × 100%. Illustrated is a representative of at least three separate experiments and the data were represented as mean ± SD. **p < .01 versus control.

MDCK cells were incubated with various doses of COM crystals for 48 h, and then subjected to Annexin V/PI staining. The cell death rate was analyzed using flow cytometry to examine the effects of differential doses of COM crystals. The data showed that the percentage of cell death increased by 0.85%, 1.8%, 6.21%, and 12.21%, respectively, at the concentration of 0.1, 0.25, 1, and 5 mM of COM crystal treatment compared with the control group cells (Figure 1). Based on these data, the concentration of 1 mM COM crystals was used for subsequent experiments of signaling pathway regulation.

### ROS generation stimulated by COM crystals

**Figure 2.**
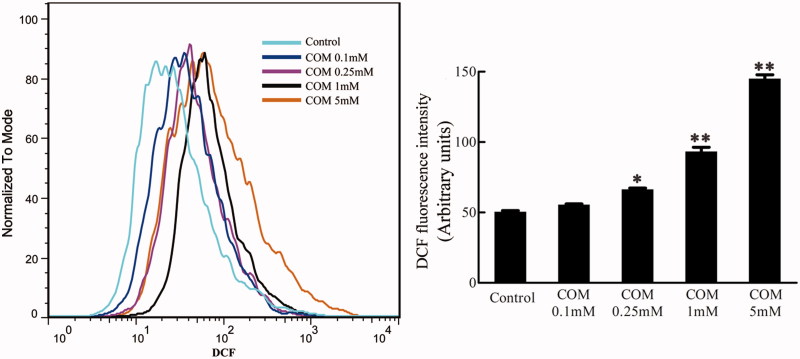
ROS generation stimulated by COM crystals. MDCK cells were treated with various doses of COM crystals for 48 h, and DCFH-DA assay using flow cytometry was performed to determine intracellular ROS. Illustrated is a representative of three separate experiments and the quantifications of data were represented as mean ± SD on the right panel. **p < .01 versus control; *p < .05 versus control.

Oxidative stress has been reported to be involved in cell injury and tight junction disruption.^33^ The DCFH-DA assay was performed using flow cytometry to evaluate the production of ROS after MDCK cells were treated with differential doses of COM crystals (0.1, 0.25, 1 and 5 mM) for 48 h. The results were represented by mean DCFH-DA fluorescence. The data showed that the generation of ROS increased in a dose-dependent manner in COM crystals-treated cells (Figure 2).

### Effects of COM crystals on tight junctions in MDCK cells

**Figure 3.**
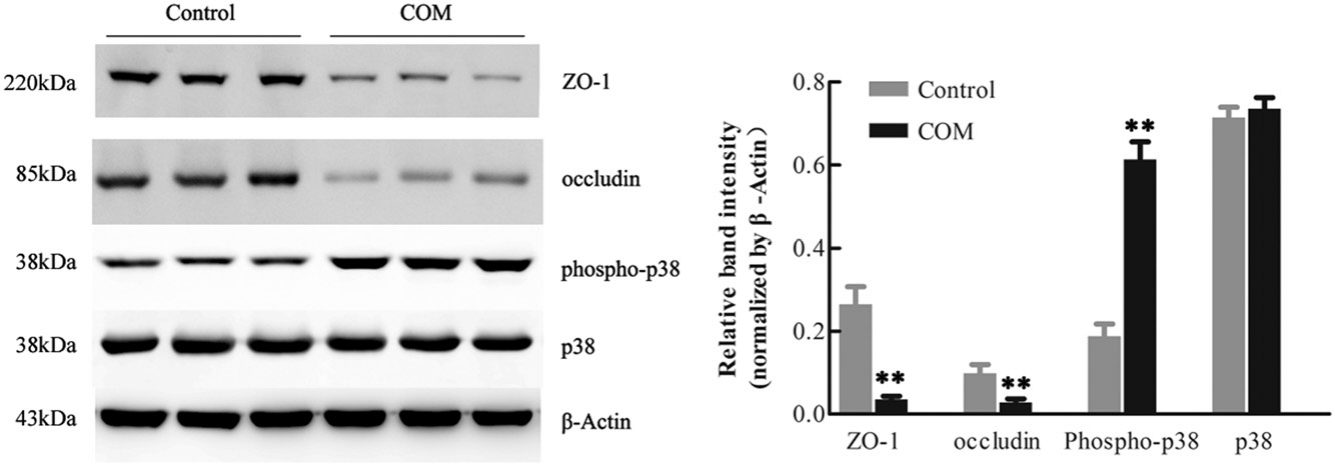
Down-regulation of tight junction related proteins in MDCK cells by COM crystals treatment. Protein levels were detected by Western blot after MDCK cells were treated with or without 1 mM COM crystals for 48 h. The quantifications of data were represented as mean ± SD on the right panel. **p < .01 versus control.

To investigate the effects of COM crystals on the expression and distribution pattern of tight junction proteins, MDCK cells were incubated with 1 mM COM crystals for 48 h. Western blot analysis showed that the expression of ZO-1 and occludin was significantly down-regulated after COM crystals exposure (Figure 3). The expression of phospho-p38 was dramatically increased by COM crystals treatment, which was consistent with the results in other study (Figure 3).^10^ In addition, immunofluorescence results showed that COM crystals treatment disrupted honey comb appearance of ZO-1 accompanied with the redistribution and dissociation of the tight junction protein in MDCK cells (Figure 6).

### ROS are essential for the regulation of COM crystals-induced disruption of tight junction

**Figure 4.**
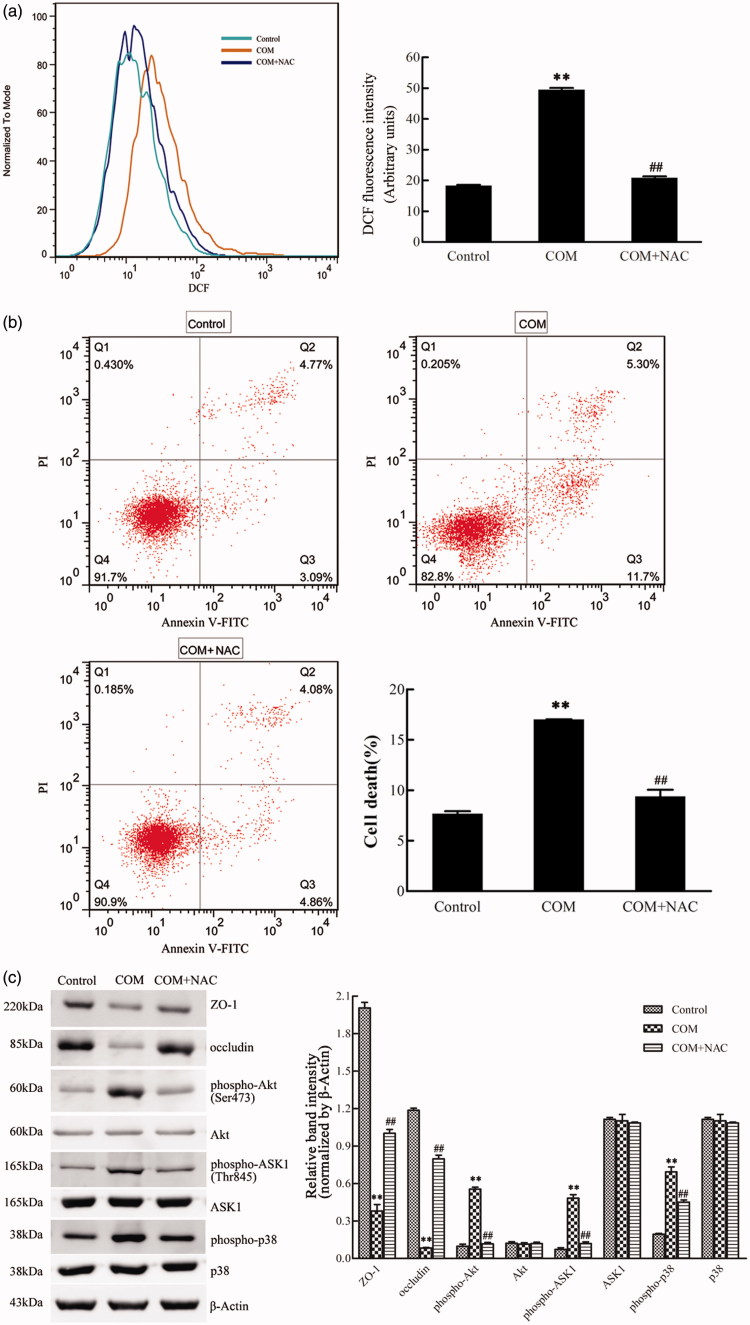
ROS are involved in the COM crystal-induced tight junction disruption. MDCK cells were pretreated with 10 mM NAC for 2 h and then incubated with 1 mM COM crystals for 48 h. (a) ROS production induced by COM was inhibited by NAC. Intracellular ROS were determined by DCFH-DA assay using flow cytometry. (b) The apoptosis induced by COM crystals was alleviated by NAC. MDCK cells treated with or without NAC were detected using flow cytometry by Annexin-V/PI staining. (c) NAC treatment inhibited the down-regulation of ZO-1 and occludin induced by COM crystal, and repressed the phosphorylation of Akt, ASK1, and p38. Protein levels were detected by Western blot and the relative band intensities were analyzed by Image-Pro Plus 6.0. Illustrated is a representative of three separate experiments and the quantifications of data were represented as mean ± SD on the right panel. **p < .01 versus control; ##*p* < .01 versus COM.

It has been demonstrated that COM crystals caused disruption and defective barrier and fence functions of tight junction of distal renal tubular epithelial cells through activating p38 MAPK.^10^ But, little is known about the upstream signaling involved in COM crystals-induced disruption of tight junction. Previous studies suggested that ROS contributed to cell apoptosis stimulated by various stimuli by activating p38 MAPK. Recently, oxidative stress induced by ROS was reported to be able to cause the disruption of tight junctions and increase of paracellular permeability in a variety of epithelial tissues.^16–18^ Therefore, we hypothesized that ROS might be involved in regulation of the disruption of tight junction induced by COM crystals. NAC, an inhibitor of ROS, was employed to evaluate whether ROS played an important role in regulation of COM crystal-induced tight junction disruption. MDCK cells were pre-incubated with 10 mM NAC for 2 h, and then incubated with 1 mM COM crystals for 48 h. Flow cytometry analysis showed that there was a significant decrease of ROS by NAC pretreatment (Figure 4(a)). Annexin V/PI staining results indicated that NAC substantially ameliorated cell death caused by COM crystals treatment (Figure 4(b)). Western blot analysis revealed that COM crystals significantly repressed the expression of ZO-1 and occludin, whereas the two tight junction proteins sustained almost unchangeable in cells pretreated with NAC (Figure 4(c)). Meanwhile, treatment of NAC attenuated the phosphorylation of Akt (Ser473), ASK1 (Thr838), and downstream p38 MAPK (Figure 4(c)) in MDCK cells. Furthermore, immunofluorescence study showed that pretreatment with NAC could preserve tight junction structure, honey comb appearance of expression pattern of ZO-1 (Figure 6). Accordingly, these observations suggested that ROS were crucial signaling molecules upstream of p38 MAPK in regulation of COM crystals-induced disruption of tight junction in MDCK cells.

### Akt is activated and involved in p38 MAPK signaling pathway in COM crystals-induced disruption of tight junction

**Figure 5.**
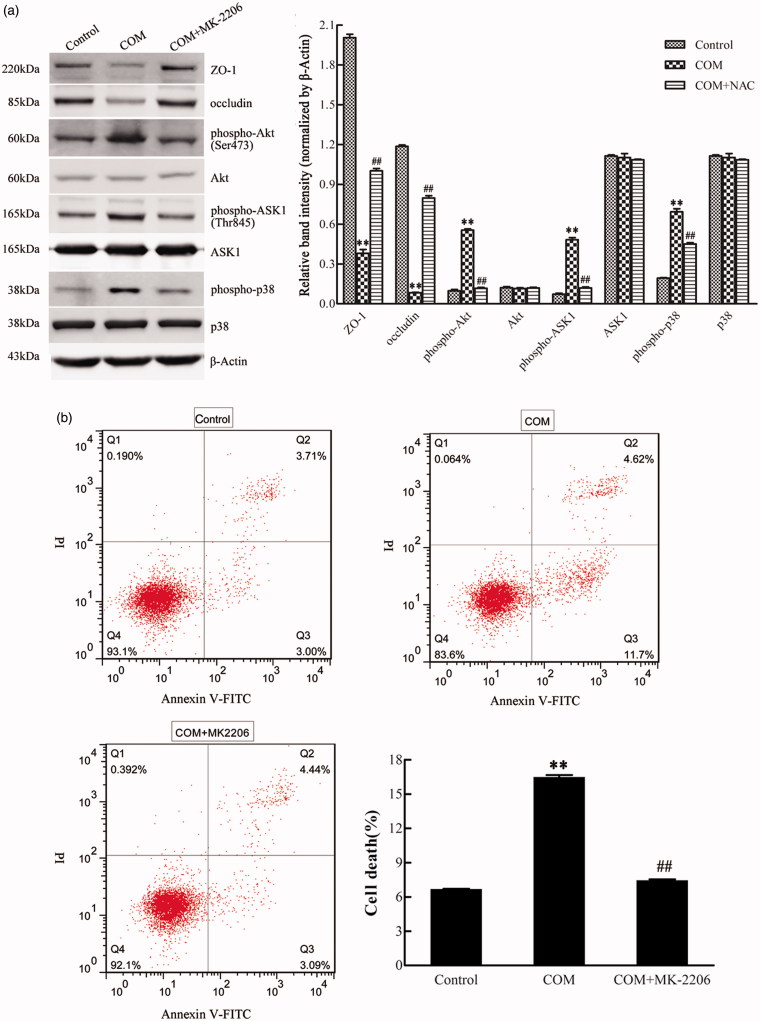
Akt is involved in COM crystals-induced tight junction disruption by activating ASK1 and p38. (a) Akt inhibitor MK-2206 alleviated the down-regulation of ZO-1, occludin, and the activation of ASK1 and p38 induced by COM crystals. Protein levels were detected by Western blot and the band intensities were analyzed by Image-Pro Plus 6.0. (b) Akt inhibitor MK-2206 inhibited the apoptosis induced by COM crystals. MDCK cells were pretreated with or without 5 μM MK-2206 for 24 h, then were incubated with 1 mM COM crystals for 48 h. Cell apoptosis was analyzed using flow cytometry by Annexin V-PI double staining. Representative data from repeated experiments performed in triplicate were presented on the right panel. **p < .01 versus control; ##*p* < .01 versus COM.

**Figure 6.**
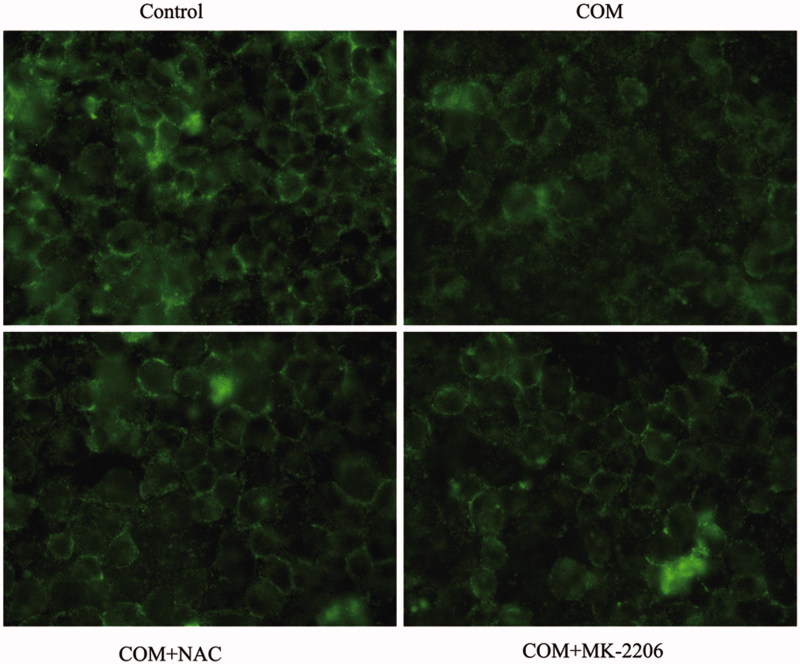
Inhibition of ROS or Akt effectively prevents the redistribution and dissociation of tight junction proteins induced by COM crystals in MDCK cells. MDCK cells were incubated with COM crystals for 48 h after pretreated with 10 mM NAC for 2 h or 5 μM MK-2206 for 24 h. Then, the cells were subjected to immunofluorescence analysis for ZO-1 using AlexaFluor-488-conjugated secondary antibody. MDCK cells grown in COM-free medium served as the control group. Original magnification was 400× for all panels.

Activated Akt kinases can trigger the phosphorylation of a number of pro-apoptotic proteins to regulate cell growth, proliferation, survival, and other cellular activities. Moreover, it has been reported that ASK1 can be phosphorylated at Thr838 directly by Akt kinases to prompt cell apoptosis.^20^ By Western blot, we observed a pronounced activation of Akt in MDCK cells exposed to COM crystals (Figure 5(a)). To obtain direct evidence for the involvement of Akt in p38 MAPK pathway in COM crystals induced disruption of tight junction, Akt inhibitor of MK-2206 was employed to address the responses of MDCK cells to COM crystals treatment. Western blot results showed that 5 μM MK-2206 significantly inhibited the activation of Akt, and the expression of ZO-1 and occludin was distinctly down-regulated (Figure 5(a)). The phosphorylation of ASK1 (Thr838) and p38 (Tyr182) was also attenuated compared with COM crystals treated cells (Figure 5(a)). Moreover, flow cytometry assay showed that inhibition of Akt by MK-2206 greatly depressed cell apoptosis (Figure 5(b)). Furthermore, the redistribution and dissociation of ZO-1 caused by COM crystals exposure were also alleviated by inhibition of Akt (Figure 6). Taken together, these data suggested that Akt played a crucial role in ASK1/p38 MAPK signaling pathway in the regulation of disruption of tight junction induced by COM crystals.

## Discussion

Tight junction is the intercellular junction that localizes between apical and basolateral membranes of epithelial cells. This junction is composed of a protein complex mainly comprising transmembrane proteins occludin and claudin, both of which form the homophilic adhesion at the extracellular region, and cytoplasmic proteins Zonula occludens (ZO-1, ZO-2, and ZO-3) binding to the intracellular region of occludin and claudin and acting as a scaffold protein along with actin filaments to provide stability to adhesion.^34^ There are two main functions of tight junction, barrier and fence functions. As a paracellular barrier, tight junction regulates and limits the passage of water, ions, macromolecules and pathogens through paracellular route; As a fence, tight junction separates apical from basolateral membranes of epithelial cells to maintain cell polarity.^35^ Dissociation of the protein complex or down-regulation of some proteins will lead to disruption of tight junction, which will further lead to infection and inflammation.^36^

Tight junction can be disrupted by various stimuli, including oxidative stress,^18^^,^^33^ pathogens^37^ and proinflammatory cytokines.^38^ ZO-1 and occludin are commonly used to demonstrate the disruption of tight junction. A previous study has shown that COM crystals can cause disruption of tight junction in renal tubular epithelial cell, accompanied with impairment of its barrier and fence functions. Adhesion of COM crystals onto renal tubular epithelial cell surface that initiates renal tubular epithelial injury is a crucial mechanism for kidney stone formation. It is observed that increased COM crystals bind to injured renal tubular epithelial cells, which lead to the crystals retention.^39^ Intratubular retention of crystals is considered as a pathological step that ultimately leads to stone formation in the kidney.^40^

Unfortunately, little is known about the involved signaling pathway and the molecular mechanisms underlying COM crystal-induced disruption of tight junction. It has been confirmed that p38 MAPK is activated in COM crystal-induced tight junction disruption in MDCK cells.^10^ IL-2R signaling was also reported to be involved in oxalate-induced tight junction disruption in a p38 MAPK-dependent manner in HK-2 cells, a line of human renal epithelial cells.^41^ Even though p38 MAPK is confirmed to be essential for the regulation of COM crystal-induced tight junction disruption, the signaling pathway upstream of p38 MAPK involved in tight junction disruption was poorly understood. In our study, it was addressed that ROS/AKT/p38 MAPK signaling pathway was activated in COM crystal-induced tight junction disruption in MDCK cells, which may provide the key to the unlocking novel biochemical mechanism in kidney stone disease. The appropriate dose and condition of COM crystal treatment that could be used to address the effects of COM crystals on tight junction without severe cytotoxicity was determined through Annexin V/PI apoptosis analysis. Based on the data in Figure 1, the dosage of 1 mM was used for subsequent signaling pathway analysis because the defect in tight junction was clearly demonstrated without significant change in cell death ratio. The increased ROS production, decreased protein expression, redistribution and dissociation of occludin and ZO-1 were observed upon COM crystal exposure in MDCK cells, which was consistent with other’s study.^9^

Then, we examined whether ROS were essential for COM crystal-induced tight junction disruption in MDCK cells. ROS are generated in various biological systems and play crucial roles in inflammation, carcinogenesis, cell apoptosis, and cellular senescence. An aberrant increase of ROS can cause alterations in cellular adenosine triphosphate and Ca^2+^ levels, which leads to mitochondrion-activation-mediated apoptotic cell death.^42^ Besides, it has been proved that oxidative stress induced by ROS is able to disrupt the epithelial tight junction. Furthermore, clinical investigations have confirmed that ROS were important molecular modulators of calcium oxalate kidney stone formation.^43^ NAC, a general antioxidant, was used as ROS scavenger to determine whether ROS were essential for COM crystal-induced tight junction disruption in our study. Western blot analysis showed that the expression of occludin and ZO-1 were significantly decreased, whereas phospho-p38 were greatly increased in COM crystals-treated cells compared with control group cells (Figure 5(a)). Redistribution and dissociation of ZO-1 induced by COM crystals were also demonstrated by immunofluorescence (Figure 6). Moreover, the downstream signals of ROS, Akt and ASK1 were determined by Western blot. The data showed that phospho-Akt (Ser473) and phospho-ASK1 (Thr838) were both up-regulated in COM crystal-treated cells compared with control cells, but the expression of total Akt or ASK1 remained virtually unchanged. However, these properties induced by COM crystals in MDCK cells were successfully abolished by the pretreatment of NAC. Collectively, these data suggested that ROS were important signal molecules upstream of p38 MAPK in regulation of COM crystal-induced tight junction disruption in renal tubular epithetical cells. Growing evidence has indicated the involvement of ROS in these pathways.^44^ For instance, ROS mediate PI3K/Akt cascade and apoptosis induced by FasL.^45^ ROS were also proved to be involved in LPS-induced activation of ASK1/p38 pathway.^46^ Interestingly, recent studies disclosed that peroxynitrite, hypochlorous acid, and 4-hydroxy-2-nonenal played key roles in barrier dysfunction in epithelial monolayer, although hydrogen peroxide is the major member of ROS that are involved in tight junction disruption.^47^

Several lines of evidence indicated the involvement of Akt in the regulation of disruption of tight junction. For example, PI3K/Akt signaling was involved in the reduced expression of tight junction proteins triggered by treatment with HIV-1 Tat protein^48^ and TGFβ1.^49^^,^^50^ ROS-induced brain endothelial tight junction dynamics also was mediated by Akt.^51^ In the present study, the Akt inhibitor MK-2206 was used to address the regulation of Akt on COM crystals-induced tight junction disruption. The data showed that the inhibition of Akt attenuated the phosphorylation of p38 and the down-regulation of occludin and ZO-1 induced by COM crystals-treatment. As shown in Figure 6, redistribution and dissociation of tight junction proteins ZO-1 were also alleviated by pretreatment with MK-2206 compared with COM crystal-treated cells. Moreover, elevated levels of ROS could increase the activity of Akt, and activated Akt in turn increases ROS generation mainly by increasing oxygen consumption and inhibiting the expression of ROS-scavengers downstream of FoxO,^51^ which indicated that ROS might induce a cycle of sustained Akt activation concomitant with a sustained increase in intracellular ROS level. Considering these results, we speculated that phospho-Akt activated p38 MAPK following ROS stimulation in COM crystal-induced tight junction disruption in MDCK cells. Therefore, our results indicated that Akt activation promoted the disruption of tight junction, which seemed to be contrary to its survival-promoting role. Recent studies showed that hyperactivated Akt increased the oxidative stress and rendered cells susceptible to ROS-triggered cell death or senescence.^20^^,^^26^ Thus, it is likely that the role of Akt is a double-edged sword and its pro-apoptotic role may be owing to the ability of increasing ROS generation and scavenging of antioxidant.

ASK1, a member of MAPKKK family member, phosphorylates and activates mitogen-activated protein kinase kinase 3 (MKK3) or MKK6, which then induces p38 kinase activities to trigger cell apoptosis. Moreover, there are two phosphorylation sites for ASK1, Thr838 and Thr83. Phosphorylation at Thr838 promotes, while phosphorylation at Thr83 negatively regulates the activation of ASK1.^20^^,^^27^ We found in our study that phospho-ASK1 (Thr838) was up-regulated by COM crystals treatment and the up-regulation was distinctly attenuated by the inhibitor of ROS or Akt accompanied with the down-regulation of phospho-p38 MAPK, which suggested that ASK1 would be a direct connection between Akt and p38 MAPK signaling. Recent studies have addressed that phos-phatidylserine exposing on the surface of cell membrane in apoptotic cells facilitated the attachment of COM crystals to the injured renal collecting duct cells,^39^ which indicated that cell apoptosis accelerated the stone formation. However, we found that neither NAC nor MK-2206 could completely ameliorate the disruption of tight junction induced by COM crystals, which suggested that there were other factors or signaling pathways contributing to COM crystals-induced tight junction disruption. For instance, it has been reported that disruption of airway epithelial tight junctions induced by oxidative stress was mediated through TRPM2-PLCγ1-PKC signaling pathway.^52^ Tyrosine kinase, Akt, and NFkB signaling were also reported to be involved in TNF-α-induced intestinal epithelial tight junction barrier impairment.^38^ Thus, disruption of epithelial tight junction is a complicated process, much more mechanisms are need to be explored in future.

## Conclusions

**Figure 7.**
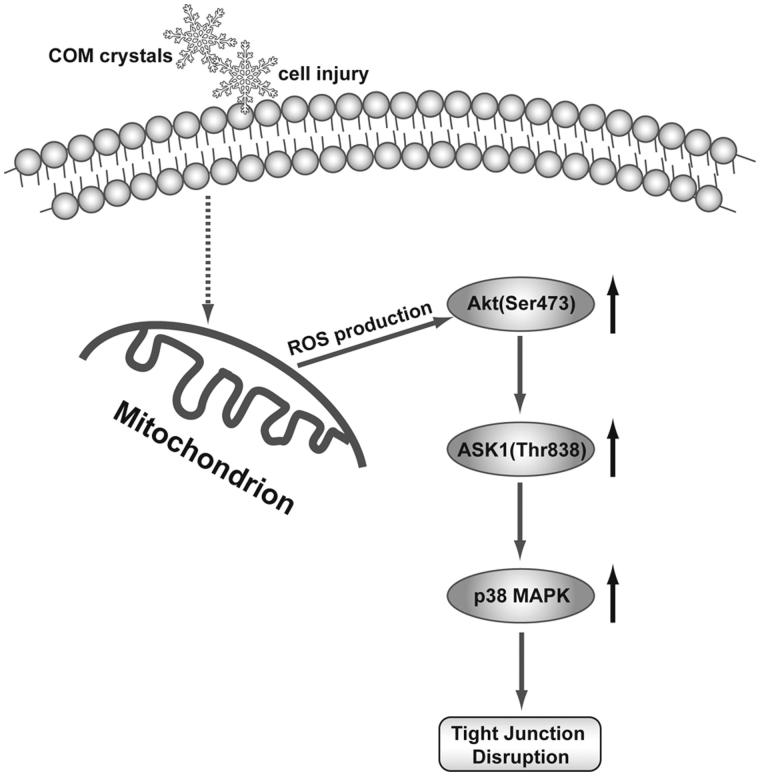
Schematic diagram of ROS/Akt/p38 MAPK signaling pathway in COM crystals-induced tight junction disruption. COM crystals stimulate the generation of ROS, which activate the phosphorylation of Akt. Akt in turn activates ASK1 by phosphorylating ASK1 on Thr838 that further triggers p38 MAPK activation, leading to the disruption of tight junction in MDCK cells.

In summary, the present study demonstrated that the substantial increase of ROS generation stimulated by COM crystal activated Akt/ASK1/p38 MAPK signaling pathway to induce tight junction disruption in MDCK cell (Figure 7). Considering the importance of tight junction disruption in injury of renal tubular epithelial cell, understanding such mechanisms in oxidative stress-induced disruption of epithelial barrier functions is likely to provide new insights into kidney stone disease, and may form a basis for the new design of treatment strategies.
